# On the Physiological Action of the Muscles Concerned in the Movements of the Lower Jaw

**Published:** 1868-04

**Authors:** 


### ON THE PHYSIOLOGICAL ACTION OF THE MUSCLES CONCERNED IN THE MOVEMENTS OF THE LOWER JAW.

Continued from page 109.

The service supposed to be rendered by the foregoing muscles is shown by the following selections:

J. Cruveilhier says, "The *sterno-hyoid*, the *omo-hyoid*, the *sterno-thyroid*, and the *thyro-hyoid* are the simplest in their structure and the simplest in their action; all coöperate to the lowering of the jaw. Moreover, if the jaw is fixed, they flex the head."*

Sappey says: "The *genio-hyoid* is the elevator of the hyoid bone when the jaw is fixed; lowerer of the jaw if the hyoid bone is motionless; flexor of the head when the hyoid bone and jaw are both fixed."†

J. Cruveilhier says of the digastric: "If the hyoid bone is fixed the posterior belly becomes the lowerer of the jaw, in consequence of the reflection of the muscle; the anterior and posterior bellies can throw the head backward."‡

Jamain says: "If the hyoid bone is fixed, the digastric coöperates in lowering the jaw."||

Todd's *Cyclopædia* says: "When the hyoid bone is fixed by its depressors, and perhaps in some degree retracted by the joint actions of the posterior belly of the digastric and of the omo-hyoid, the anterior belly, both passively as a reflected cord, and actively in virtue of its muscular fibres, depresses the lower jaw, and opens the mouth."*

"Chief action of the omo-hyoids is to tighten the cervical fascia during deglutition; they are also capable of depressing the hyoid bone."†

---

*Traite d'Anatomie Descriptive. 3me ed., tome 2me, p. 179. Paris, 1851.

†Traite d'Anatomie Descriptive. Tome 1, premiere partie, p. 213. Paris. 1850.

‡Traite d'Anatomie Descriptive. Troisieme edit, tome deuxieme. p. 182. Paris. 1851.

||Nouveau Traite Elementaire d'Anatomies Descriptive. p. 180. Paris. 1853.

*Todd's Cyclopædia. Vol. iii, p. 564.

†Ibid. p. 563.

Gray's Anatomy of the digastric, mylo-hyoid, and genio-hyoid: "When the hyoid bone is fixed by its depressors and those of the larynx, they depress the lower jaw;"‡ and further, that in deglutition "the anterior belly of the digastric carries the hyoid bone, &c., upward and forward and the posterior belly upward and backward," and says of the *platysma-myoides:* "Its anterior portion, the thickest part of the muscle, depresses the lower jaw."||

Henle thinks: "The platysma-myoides are not depressors of the lower jaw."§

Duchenne says: "Its action being exhausted by the mobility of the integuments of the face, the neck and the chest, it has no longer sufficient strength to depress the lower jaw."¶

Cruveilheir says: "The platysmas are sometimes unequal in strength."**

Ziemssen says: "The muscle is sometimes absent."††

The absence of the platysma-myoides in some cases, and its inequality in others, proves that it is not of any consequence in depressing the jaw, which is a movement requiring great promptness and exactitude. It may be held between the thumb and finger, near the front of the jaw, and if care is taken to discriminate between it and the integument, it may be felt that the muscle pays no attention to the movement of the jaw.

The muscles which centre in the hyoid bone have power to control and move the organs to which they are attached, (and of which they are in several instances important parts,) subject, however, to the following limitations. The *stylo-hyoid ligament* passes down on each side from the styloid process of

---

‡Gray's Anatomy. 2d American Edition. p. 260.

||Ibid. p. 256.

§Handbuch der Muskellehre des Menschen, von Dr. T. Henle, Professor der Anatomie in Goettingen. p. 108.

¶De l'Electrisation Localisee. p. 380. Paris, 1858.

**Traite d'Anatomie Descriptive. Troisieme edit., tome deuxieme. p. 166. Paris, 1851.

††Die Electricitæt in der Medicin, p. 44. Berlin, 1857.

the temporal bone to the little horn of the hyoid bone. These ligaments have, therefore, a slanting course, while the *supra-hyoid aponeurotic layer*, between the hyoid bone and the inner side of the front of the jaw, has a horizontal direction. By this arrangement the glottis and its covering, &c., are held at some distance from the back of the pharynx, and free respiration secured, independent of muscular action, while the hyoid bone can move upward or forward to a considerable extent, and be returned to its natural position when at rest, in any direction that is not back of this resting-place. But below this the downward, and especially the backward movements of the hyoid bone are very limited, being only what is gained by the tightening of the ligaments, &c.

Although a case is sometimes seen in which the *stylo-hyoid* ligaments give place to muscles, in others they are entirely ossified, and the temporal bones and the hyoid bone are in one piece. Showing that, depression of the hyoid below its natural position when at rest, is unnecessary, except to a trifling extent.

The digastric muscle is set down as drawing the hyoid bone backward and forward in deglutition, and as depressing the jaw by acting as " a reflected cord." These services are inconsistent with each other and with the anatomy of the parts. If it were fixed so as to draw the bone backward and forward, it could not slide and be of service as a " reflected cord " sufficiently to lower the jaw. To do the latter the anterior belly should be inserted higher up the jaw, while a long unrestricted tendon of the muscle should run through a fixed loop on the lower border of the hyoid bone, which last should also be freed from the styloid ligaments, and be drawn down half-way to the sternum every time the jaw opened wide, and proportionally for less opening.

In respect to the united action of both bellies drawing the head backward, it is only necessary to say that the origin of the digastric is partly in front of a line drawn across, just behind the condyles of the occipital bone; it could not, therefore, draw the head back appreciably even if its insertion were directly under its origin. It is consequently a mistake to suppose it can do so when its direction forward is more horizontal than vertical. In fact this muscle is the great agent drawing the head *forward.* The posterior belly slants down to the hyoid bone, but the anterior is nearly horizontal in its course, and when the muscle acts it tends to the line of its attachments by drawing or endeavoring to draw the hyoid bone upward unless the jaw is much depressed, when, as the muscle is straight, or nearly so, it has no power to raise the hyoid bone. But in several important services the digastric acts in concert with the omo-hyoid. In this way the muscles passing from the hyoid bone to the front of the jaw, including the anterior belly of the digastric, are as effectually antagonized as if a powerful muscle passed from each side of the hyoid bone to the opposite cervical vertebræ, with the advantage of greater length of muscle to contract, and easier adaptation to the movements of the jaw; and the muscles in front of the hyoid bone act, when necessary, in alternation with the omo-hyoid and the posterior belly of the digastric. More frequently, however, the anterior belly of the digastric acts with the posterior belly and the omo-hyoid, for they keep the head upright. In doing this the omo-hyoid muscle and the posterior belly of the digastric draw or hold the hyoid bone back, while the anterior belly of the digastric brings in the chin, and the *temporal* and other elevators of the jaw draw the head forward; in this way the digastric acts on a long lever, as the head rocks on a centre, but a little below the entrance of the external ear. The digastric and omo-hyoid muscles are always active during forward or backward movements of the body or head. They do for the head what the sterno-mastoid muscles do for the spine, and their action can be felt easily with the finger, in sitting down or rising up, &c. They are also powerful rotators of the head, and the action of the omo-hyoid is singularly quick in sudden turns of the head, (as with the sterno-mastoid muscles,) the digastric being useful in assisting to keep the hyoid bone up in place, it being held laterally by the apo neurosis and probably by the mylo-hyoid muscle.

If the end of the finger is placed just behind the origin of the *cleido-mastoid* during these movements, the omo-hyoid will be felt rising above the clavicle, and carrying the cervical fascia upward or backward; and if a finger is placed behind the mastoid process so as to cover the end of the digastric notch, the digastric muscle will be felt acting in concert with the omo-hyoid, and the anterior belly can be felt between the jaw and hyoid bone. The peculiar attachment of the digastric can now be appreciated, as the hyoid bone is left sufficiently free in its various movements, although it is at the same time the center of control and support to the head. The importance of this support to the head can hardly be over-estimated, for the weight of the head beyond the atlas must be balanced. This the digastric and omo-hyoid muscles do effectually by acting upon the jaw, which is a lever whose length below the top of the atlas is over one-third of the height of the head above the atlas. The points from which these muscles act are the mastoid process and the shoulder; the vertex of their angle being in the hyoid bone, from whence they draw in the chin; in this direction they are very active and powerful. They not only balance the head in locomotion and leave the other muscles free to act in deglutition, vocalization, and articulation, but frequently coöperate with them.

The following quotations show the opinions entertained as to the action of the muscles which move the lower jaw:

Gray's *Anatomy* says: "The temporal masseter and internal pterygoid raise the lower jaw against the upper with great force. The two latter muscles, from the obliquity in the direction of their fibres, assist the external pterygoid in drawing the lower jaw forward upon the upper, the jaw being drawn back again by the deep fibres of the masseter, and posterior fibres of the temporal. The external pterygoid muscles are the direct agents in the trituration of the food, drawing the lower jaw directly forward, so as to make the lower jaw project beyond the upper. If the muscle of one side acts, the corresponding side of the jaw is drawn forward, and the other condyle remaining fixed, the symphysis deviates to the opposite side. The alternation of these movements on the two sides produces trituration."*

Todd & Bowman's *Physiological Anatomy*, part iii, p. 539, says: "The *external pterygoid* neither raises nor depresses the lower jaw."

My own views as to these muscles also, differ materially on some points from those expressed in the quotations. The *stylo-hyoid ligaments* make it impossible that the hyoid system of muscles can depress the lower jaw by acting upon it vertically, and the following experiments show that they do not.

By resting the finger on the thyroid cartilage, with its end placed against the hyoid bone, it will be found that they do not descend when the jaw is opened.† Further, if the thumb is placed under the chin and the jaw held *firmly* open against it, by carefully throwing the hyoid bone up by swallowing, it will be found that the jaw is held down by other muscles than those inserted into the hyoid bone; as it will not go up if the experiment is properly conducted, even when the hyoid bone is carried above the border of the jaw. This may, however, require some care and practice; as the jaw is, in general, nearly shut in deglutition, and its depressors are inclined to relax in sympathy with the movements of the other parts. The position of the *masseter* is too well known to need particular description. The fibres of the deep portion have a more perpendicular direction than those of the superficial portion, the last passing backward as much as downward. The deep portion draws the jaw upward and backward, the superficial portion upward and forward. The *internal pterygoid* has the same general direction as the superficial portion of the *masseter*, excepting that as it arises from the pterygoid fossa it passes considerably outward to reach its insertion on the inner side of

*Gray's Anatomy, Descriptive and Surgical. 2d American Edition, p. 252. Phila. 1865.

†Except to a trifling extent, when the jaw is opened unusually quick or wide.

the ramus and angle of the jaw. It has, therefore, not only the upward and forward motion of the superficial portion of the masseter, but also lateral power over the jaw.

These muscles not only raise the jaw and teeth, when cutting with the incisors and crushing with the molars, but are also the main movers of the jaw in trituration. In cutting they bring the jaw forward bodily; in triturating they exert more foward and lateral action on one side than on the other; by this the jaw is thrown over to the opposite side and then *drawn in and carried out continuously on that side*, and not carried over to the other side. It is a mistake to suppose that triturition of the food is effected by the alternate action of the muscles of both sides. When the jaw and teeth are perfect, the teeth of one side are only used, until the muscles tire, perhaps, and then those of the other side are resorted to. If the teeth are tender, badly placed, or deficient on one side, trituration is performed on the other only.

The *temporal* muscle, which covers so large a portion of the side of the head, and is strongly inserted into the inner surface, apex and anterior border of the coronoid process of the jaw, pulls its insertion upward and backward, and assists the *masseter* and *internal pterygoid* muscles in cutting, crushing and triturating the food.

The *external pterygoid* is a short, thick muscle, which arises from the pterygoid ridge on the great wing of the sphenoid, and the portion of bone included between it and the base of the pterygoid process, from the outer surface of the external pterygoid plate and the tuberosity of the palate and superior maxillary bones. It arises in two portions separated by a short interval; they both pass outward and backward and are inserted into a depression in front of the condyle of the lower jaw, and into the corresponding part of the inter-articular fibro-cartilage. The separate portions join and form one muscle previous to their insertion; the middle fibres being horizontal, but as the origin of the muscle is very wide vertically, the upper portion descends in passing back, while the lower ascends. This strong and beautiful muscle has, from its peculiar situation, great influence over the jaw. The origin being more internal than the insertion, gives the muscle control over the condyle laterally, by which it is held firmly in the glenoid cavity, and the great strength of the muscle tends to keep the condyle from being driven back by falls, or blows upon the chin, which otherwise might occur easily, as the glenoid fossa is very shallow behind.

A more prominent service of this muscle is when it brings the condyle forward either on its own side alone, as in trituration, or with its fellow of the opposite side, as in cutting by the incisors. In these movements it acts in concert with other motors of the jaw. To consider it especially the triturating muscle is no more correct than to suppose that when triturating with a pestle in a mortar, the thumb and forefinger (which are further from the grinding surface) are the triturators rather than the fingers below.

The *external pterygoid* muscle controls the upper end of the jaw in concert with the *temporal*, while the muscles attached to the body have especial control below, and by the concerted action of all, power and steadiness are secured in the complicated movements of the jaw, and of the lower teeth against the upper. The *external pterygoid* muscle has one office peculiar to itself, that of holding the condyle fixed on any part of the eminentia articularis, to which it may be drawn in movements of the jaw. For this service the muscle is admirably fitted by the great width of its origin, which enables it to brace the condyle so firmly against the part above and in front of it, that the jaw is fixed, even when wide open, as firmly as if the condyle were hinged in that position.

The *eminentia articularis*, the rounded projection which forms the front of the glenoid fossa, is indispensable to this fixation of the condyle; and as the condyle is coming forward mounts this eminence, the jaw, while going down in front, is also carried down bodily, by which the back teeth of the lower and upper jaws are widely separated. These advantages are gained while the center, or rather the curve, upon which the jaw turns, is three-fifths down toward the angle. This may be tested by placing the wrist and hand around the back of the neck and applying the end of the middle finger to the back of the ramus, and finding the point upon which it turns, which is probably near the insertion of the internal lateral ligament. By this arrangement the jaw is opened promptly and widely, without interfering with the parts behind; which would be injuriously pressed upon if the condyle remained still and the angle went back far enough to open the jaw wide.

The *external pterygoid* is now to be spoken of in a service in which the action of the muscle is greater in range, frequency and importance than in any other, and in which it has never before been recognized, *that of opening the mouth.* This muscle antagonizes the temporal, masseter and internal pterygoid muscles, and is, especially by its lower head, *the depressor of the lower jaw.* It does this alone when necessary, without assistance from any other muscle. Its ability to do this may be proved by bracing the arm against the breast and applying the thumb firmly to the chin; or better, by placing the jaw flat on the mantel, or any fixed support, then on opening the mouth the head will go backward, being drawn back by the external pterygoids whose insertions in the condyles are fixed by support of the jaw.

The action of the external pterygoid muscles in opening the jaw is similar to their action when the jaw is brought forward to cut with the incisors, the difference in effect being produced by other muscles; the temporals probably act similarly in both movements. The masseters and internal pterygoids, however, must relax in the opening of the jaw instead of assisting to carry it forward as in cutting, while the digastrics, which relax in the forward movement of the jaw, undoubtedly assist to draw the chin back in gaping, vomiting, and in very wide opening of the mouth when voluntary. When not otherwise employed, it is probable that they always assist in opening the jaw, as they are admirably fitted for carrying the chin back, when the condyle is going forward. It is impossible that they could carry it back readily if the condyles were not at the same time pulled forward. For, when the chin points down very much (as in some persons even with the jaw closed), the digastric muscle forms but a slight angle at the loop, and decided action of it in such cases would tend to keep the condyle of the jaw back in the glenoid cavity from the start, or soon after, as the opening of the jaw would straighten the digastric so much that it would pull nearly in the direction of the ear and have but little power to carry the chin back. That the digastrics draw the condyles of the jaw back in the glenoid fossæ when acting without decided action of the external pterygoids, is demonstrated by exerting strong suction with the tongue against the roof of the mouth. In doing this the upper and lower teeth are held a little apart and the condyle of the jaw can be felt to move back, as the muscles tighten, if the point of the little finger is pressed firmly into the ear so that its front rests against the auditory process. In cases where the anterior belly of the digastric ascends from its loop to the chin, so that it has some vertical direction to favor it; and even supposing it were assisted by strong back fibres in the mylo-hyoid muscles, it is not possible that the condyle could release itself from the *eminentia articularis*; and unless it did so and came forward, the jaw could open only to a trifling extent, for it would act as if hinged in the glenoid fossa. But with the external pterygoids drawing the condyles forward, the jaw opens readily, as upon a center in the ramus, and the question arises as to what the jaw really turns upon.

The masseter and internal pterygoid muscles hold the angle very much as if a sling were passed around it, while the *stylo-maxillary ligament* is also inserted into the angle. It might therefore be thought that the jaw turned upon the angle if this part did not go back during the forward movement of the condyle, thus proving that the jaw turns on a portion of the ramus between the angle and the condyle. Under all the circumstances the insertion of the *internal lateral ligament* around the inferior margin of the dental foramen marks the center upon which the jaw opens with sufficient exactness. This ligament is a thin aponeurotic expansion, which descends perpendicularly from the extremity of the spinous process of the sphenoid bone, and cannot properly be consided the special support of the jaw in its downward or upward movement. It is more likely that the pterygoid-maxillary ligament also exerts an influence, and that all the ligaments and muscles inserted into this part of the bone are instrumental in determining the centre upon which the jaw opens.

The shape of the jaw, whether congenital, or modified by age, or accident, may also vary the location of the center somewhat; but in every case the condyles must come forward to open the jaw effectually. This can be done only by the *external pterygoid* muscles, whose special office is to move and also fix the condyles, and in connection with the temporal and other elevators, the jaw also. In this way only can the digastrics and associate muscles act efficiently in their most important functions; otherwise they would be disturbed by undue movement of the jaw. Sometimes, however, the digastrics, as before stated, assist in moving and holding the jaw.

When the *external pterygoids* throw the jaw wide open, the chin is much below the hyoid bone, but controlled by the anterior bellies of the digastric muscles, the hyoid bone being fixed by the posterior bellies and by the *omo-hyoid* muscles, which can now be felt in action. Consequently the three extremities of the jaw, the chin, the angle, and the condyle, have each diverging or converging muscular support. The *digastrics* go to the chin, the *internal pterygoids* go to the angles, and the *external pterygoids* to the condyles. This arrangement of the muscles in connection with the ligaments holds the jaw firm in all its complicated movements.

It has been shown that the *sterno-cleido-mastoid* muscles do not rock the head, but that they give anterior support to the atlas, while the splenii muscles give posterior support, and that in combination they support it and the head laterally. This support is so complete that the head has a firm resting place, upon which it is held securely in all positions. The muscles which give form to the neck, and support and move the spine and head, are so arranged as to leave the front of the spine free for the tongue, larynx, and trachea, with their special muscles, &c., and for nerves, blood-vessels, and glands of prime necessity.

These important parts are shut in and protected by the lower jaw, which gives attachment to muscles indispensable to those organs in the performance of their functions, while its own movements are in strict subordination to them. By this respiration is secured from interruption at all times, and occasionally made tributary to vocalization and articulation, and the vocal tube modified and moved with wonderful delicacy and energy in its participation in these functions, and in accordance with those of mastication and deglutition.

The lower jaw is also the great lever by which the head is held upright.

These explanations of the physiological action of the muscles which control and influence the lower jaw, prepare the way for the diagnosis of its fractures, upon which I purpose to write hereafter.

---

We recently promised to present an article through the pages of the Register, on the subject of taking impressions; and now we hope some body will assist us, by sending us the article and we will print it; a proper division of labor is all we ask.

---

The second edition of Taft's Operative Dentistry is out, and came to hand a few days ago. T.

